# Supplementary material for: Counting young birds: A simple tool for the determination of avian population parameters
Source: PLoS One. 2023 Feb 17;18(2):e0279899. doi: 10.1371/journal.pone.0279899 (PMC9937500; doi:10.1371/journal.pone.0279899)
Supplement: S1 File — (PDF) [file pone.0279899.s001.pdf]

Supporting information S1

Counting young birds: a simple tool for the determination of avian population parameters

Werner Oldekop, Gerd Oldekop, Kai Vahldiek, Frank Klawonn, and Ursula Rinas

Content

1. Introduction ... 3
2. Background for the computations ... 4
   2.1. Table of abbreviations ... 4
   2.2. General notations and definitions ... 4
      2.2.1. Population / Number of birds “**N**” ... 4
      2.2.2. Population increase ... 5
      2.2.3. Population decrease: Mortality “**M**” ... 5
      2.2.4. Life expectancy “**L**” ... 5
      2.2.5. Cumulative mortality “**cM**” and survival probability “**S**” ... 6
      2.2.6. Yearly or relative (conditional) mortality “**rM(a)**” ... 6
      2.2.7. Population growth and growth rate “**r**” ... 6
   2.3. Basic equation for population dynamics ... 6
      2.3.1. Simplifying assumptions ... 6
      2.3.2. Example: Doubling time “**D**” ... 7
      2.3.3. Reformulations of the basic equation for population growth ... 7
   2.4. Age parameters in a population ... 7
      2.4.1. Age distribution “**P(a)**” ... 7
      2.4.2. Mean age “**A**” ... 8
   2.5. Further parameters ... 8
      2.5.1. Relative mortality in the population “**rP(a)**” ... 8
      2.5.2. Generation length “**G**” ... 8
3. Types of mortality distributions ... 9
   3.1. Degenerative mortality ... 9
   3.2. Geometric distribution (Expo) ... 10
      3.2.1. Application example 1: Average life expectancy “**L**” ... 10
      3.2.2. Application example 2: Mortality “**M(a)**”, survival probability “**S(a)**”, and age distribution “**P(a)**” .. 10
      3.2.3. Application example 3: Varying proportion of young birds ... 11
   3.3. Modified geometric mortality distribution (ModExpo) ... 11
      3.3.1. Numerical procedure ... 12
      3.3.2. Practical computation with EXCEL ... 12
   3.4. Bootstrapping and calculations based on R ... 13
      3.4.1. Background information bootstrapping ... 13
      3.4.2. Instructions for using the “ModExpo” method in R ... 13
   3.5. Other approaches and further perspectives ... 14
4. References ... 15

1. Introduction

This Supporting Information to the manuscript “Counting young birds……” describes the mathematical background for the analyses provided in the manuscript. It also explains how to apply these computations to other or new data using Excel or R.

2. Background for the computations

2.1. Table of abbreviations

Name	Interpretation	s. Section
**N**	Number of birds in a population	2.2.1
**g**	Observed proportion of young birds: $\mathbf{N_{juv}/(N_{ad}+N_{juv})}$	2.2.2
**M(a)**	Mortality per year	2.2.3
**a**	Age in years	2.2.3
**L**	Average life expectancy	2.2.4
**Le(a)**	Average residual life expectancy given the bird has reached age "**a**"	2.2.4
**cM(a)**	Cumulative mortality until age "**a**"	2.2.5
**S(a)**	Survival probability until age "**a**": (**S(a)=1-cM(a)**)	2.2.5
**rM(a)**	Relative mortality for year "**a+1**", given the bird reached age "**a**"	2.2.6
**r**	Growth rate per year	2.2.7
**D**	Doubling time	2.3.2
**P(a)**	Proportion of birds of age "**a**" in the population	2.4.1
**A**	Mean age in a population	2.4.2
**rP(a)**	Relative mortality in the population	2.5.1
**G**	Generation length	2.5.2
**B1, B2**	First and last breeding age	2.5.2
**M1**	Mortality in the first year of life	3.3
$\mathbf{a_{max}}$	Maximum (known) possible age depending on the species	3.3

2.2. General notations and definitions

In the following, the term population refers to birds of a specific species in a larger region during a certain period of time. This can encompass migratory birds encountered in their stopover places (e.g. Common Cranes from Scandinavia resting in the region of the Darß-Zingster Boddenkette and Rügen) as well as a local breeding population (e.g. local Black Redstarts). The most important parameter is the proportion "**g**" of young birds, already introduced in the main text. This implies that the average life expectancy "**L**" of a bird in a stationary population is given by

$$L=1/g \quad \text{F1}$$

The situation is more complex in non-stationary populations. Also, other parameters such as the mean age "**A**" cannot be computed easily in non-stationary populations due to the dependency on individual mortality.

2.2.1. Population / Number of birds "**N**"

The population is considered year-wise. The start point of each counting year is usually not the first of January but the month when the counting starts, e.g. during migration when young and adult birds can still be well distinguished. In the case of Common Cranes, this would be October.

The number of birds in a population in a specific year “j” (e.g. “j=2018”) is denoted by “$\mathbf{N_j}$“. Then

$N_j – N_{j-1}$	F2

is the growth of the population from year “**j-1**” to the following year “j”. A negative growth means that the population is shrinking.

2.2.2. Population increase

Given the proportion “$\mathbf{g_j}$“ of young birds in year “j”, the population increases by

$N_j * g_j$	F3

Increased mortality of young birds is not considered, when counting occurs after fledging or in a later period of time. Directly after hatching the portion of young birds in a population will be larger resulting in a lower average life expectancy in this population because of the enhanced mortality of fresh hatchlings or very young birds.

2.2.3. Population decrease: Mortality “**M**”

Mortality leads to a decrease of the population. In contrast to the proportion of young birds, mortality cannot be observed directly, but must be estimated from the counts of young and adult birds. Define the mortality as

$M_{i,j}$ = probability that a young bird born in year “i” dies in year j>i	F4

Because the counting is carried out only once – or during a defined period – per year, the death of birds dying between the counts of year “i” and year “i+1” must be assigned to either year “i” or “i+1”. Here we assign it to year “i+1”, implying that “$\mathbf{M_{i,i} = 0}$” holds. This can lead to a slight overestimation of the life expectancy.

Assuming that the mortality depends only on the age of the bird, then the probability that a bird dies at age “**a**” is denoted by

$M(a)$	F5

As explained above “**M(0) = 0**”.

2.2.4. Life expectancy “**L**”

Given the mortalities “**M(a)**” for each year, these probabilities must sum up to 1 and the average life expectancy “**L**” can be computed from the probabilities.

$\sum_{a=0}^{\infty} M(a) = 1$	F6
$L = \sum_{a=0}^{\infty} a \cdot M(a)$	F7

The residual life expectancy “**Le(a)**” of a bird that has reached already age “**a**” is given by the conditional expectation.

$Le(a) = \frac{\sum_{k=0}^{\infty} k \cdot M(a+k)}{\sum_{k=0}^{\infty} M(a+k)}$	F8

Where “**k**” corresponds to the additional years a bird of age ”**a**” still survives.

2.2.5. Cumulative mortality “**cM**” and survival probability “**S**”

The cumulative mortality “**cM**”, i.e. to die before age “**a+1**” or the probability that a bird lives for at most “**a**” years, is

$$cM(a) = \sum_{k=0}^{a} M(k) \qquad cM(0) = 0 \qquad cM(\infty) = 1 \tag{F9}$$

The survival probability “**S(a)**” is then

$$S(a) = 1 - cM(a) = 1 - \sum_{k=0}^{a} M(k) = \sum_{k=a+1}^{\infty} M(k) \qquad S(0) = 1 \qquad S(\infty) = 0 \tag{F10}$$

For “**a>0**“, we have

$$M(a) = S(a-1) - S(a) \tag{F11}$$

2.2.6. Yearly or relative (conditional) mortality “**rM(a)**”

The yearly or relative (conditional) mortality “**rM(a)**” is the probability that a bird dies at age “**a+1**” given it survived until age “**a**”.

$$rM(a) = \frac{M(a+1)}{S(a)} = \frac{S(a) - S(a+1)}{S(a)} \tag{F12}$$

2.2.7. Population growth and growth rate “**r**”

The population growth, defined in Eq. F2, which can also be negative if more birds die than are born, can be computed as the number of young birds given in Eq. F3 minus the number of old birds that die in the corresponding year.

$$N_j - N_{j-1} = g_j \cdot N_j - \sum_{(i<j)} g_i \cdot N_i \cdot M_{i,j} \tag{F13}$$

In order to avoid the absolute population numbers that are difficult to determine we consider relative quantities.

From Eq. F13, the yearly growth “**r**” rate can be inferred simply by dividing “$\mathbf{N_j\text{-}N_{j-1}}$” by the total number of birds of the population “$\mathbf{N_{j-1}}$” in year “j-1”.

$$\frac{N_j - N_{j-1}}{N_{j-1}} = r_j = g_j \cdot \frac{N_j}{N_{j-1}} - \sum_{(i<j)} g_i \cdot \frac{N_i}{N_{j-1}} \cdot M_{i,j} \tag{F14}$$

Changes of the population caused by migration can be neglected if out- and in-migration are balanced or if the observational area is sufficiently large.

2.3. Basic equation for population dynamics

2.3.1. Simplifying assumptions

Given the proportions “$\mathbf{g_i}$” of young birds and the yearly mortalities “$\mathbf{M_{i,j}}$” are known and initial values for the past years are available, Eq. F13 provides a means to compute the number “$\mathbf{N_j}$” of birds in the population for the succeeding years. Usually these parameters are unknown. But with the following simplifying assumptions one can still derive estimates for the number “$\mathbf{N_j}$”.

- The proportion "**g**" of young birds remains constant over a longer period or can be replaced by its mean.
- The growth rate "**r**" remains constant over a longer period or can be replaced by its mean.
- Mortality "$\mathbf{M_{i,j}}$" depends only on the age.

With these assumptions, Eq. F14 implies the basic equation for population growth (see also [1]).

$$\frac{N_j}{N_{j-1}} = 1 + r \qquad \frac{N_{j-a}}{N_{j-1}} = (1+r)^{1-a} \qquad \text{and} \qquad r = g\cdot(1+r) - g\cdot\sum_{a=1}^{\infty}(1+r)^{1-a}\cdot M(a) \qquad \text{F15}$$

2.3.2. Example: Doubling time "D"

Given the growth rate "**r**", the population increases after "x" years by the factor "$(1+r)^x$". In order to calculate the time that it takes until the population has doubled, "$(1+r)^x=2$" must be solved, leading to the doubling time "**D**".

$$D = \frac{\ln(2)}{\ln(1+r)} \qquad \text{F16}$$

2.3.3. Reformulations of the basic equation for population growth

Solving Eq. F15 for the proportion "**g**" of young birds leads to

$$g = \frac{r}{1 + r - \sum_{a=1}^{\infty}(1+r)^{1-a}\cdot M(a)} \qquad \text{F17}$$

and with Eq. F11 we obtain

$$g = \frac{1}{\sum_{a=0}^{\infty}(1+r)^{-a}\cdot S(a)} \qquad \text{F18}$$

The average life expectancy "**L**" is then with Eq. F7

$$L = \sum_{a=1}^{\infty} a\cdot M(a) = \sum_{a=1}^{\infty} a\cdot(S(a-1) - S(a)) = \sum_{a=0}^{\infty} S(a) \qquad \text{F19}$$

For the special case "**r=0**" Eqs. F18 and F19 imply

$$g = \frac{1}{L} \qquad \text{respectively} \qquad L = \frac{1}{g} \qquad \text{F20}$$

In a stationary population with "**r=0**" the average life expectancy "**L**" is the reciprocal of the proportion of young birds in the population "**g**" independent of all other parameters. In a shrinking or growing population "**L**" does not only depend on "**g**" but also on the mortalities. If "**r**" is small, Eq. F20 also holds approximately.

2.4. Age parameters in a population

2.4.1. Age distribution "P(a)"

The age distribution can be derived from the following thoughts concerning the population in year "j" where "**N(j)**" denotes the total number of birds in the population in year "j".

- There are "**N(j)*g**" young birds of age "**a=0**".
- There are "**N(j-1)*g* S(1)**" one year old birds "**a=1**".
- Etc. ...
- There are "**N(j-a)*g* S(a)**" birds of age "**a**".

The factor "**S(a)**" takes into account that the corresponding birds have to survive at least until age "**a**".

According to Eq. F14 we have for a constant growth rate "**r**"

$$\frac{N(j-1)}{N(j)} = \frac{1}{1+r} \quad \text{and} \quad \frac{N(j-a)}{N(j)} = (1+r)^{-a} \qquad \text{F21}$$

The proportion "**P(a)**" of age "**a**" years is then

$$P(a) = g \cdot (1+r)^{-a} \cdot S(a) \qquad \text{F22}$$

which gives the age distribution "**P(a)**" under the assumption of a constant growth rate "**r**".

We obviously have that these probabilities are normalised, i.e.

$$\sum_{a=0}^{\infty} P(a) = 1 \qquad \text{F23}$$

2.4.2. Mean age "**A**"

The mean age in the population is then

$$A = \sum_{a=0}^{\infty} a \cdot P(a) \qquad \text{F24}$$

2.5. Further parameters

2.5.1. Relative mortality in the population "**rP(a)**"

There is no unified definition of relative mortality in the population "**rP(a)**". It refers to the relative change of the number of birds for the specific age groups.

$$\text{relative mortality in age group a} \qquad rP(a) = \frac{P(a) - P(a+1)}{P(a)} \qquad \text{F25}$$

In a stationary population "**r=0**", "**rP(a)**" is equal to the mortality "**rM(a)**". However, for a non-stationary population, "**rM(a)**" referring to the probability for a bird of age "**a**" to die will not change, whereas "**rP(a)**" depends on the growth rate "**r**".

2.5.2. Generation length "**G**"

In population biology, the generation length "**G**" refers to the average time between two succeeding generations. It corresponds to the mean age of the breeding birds. Let "**B1**" denote the (mean) age when birds breed the first time and "**B2**" the (mean) age when birds breed the last time, then the generation length "**G**" is given by

$$G = \frac{\sum_{a=B1}^{B2} a \cdot P(a)}{\sum_{a=B1}^{B2} P(a)} \qquad \text{F26}$$

The numerator in Eq. F26 corresponds to the mean age of the breeding birds. The normalisation by the denominator is necessary because the breeding birds do not represent the full population.

(If the breeding success rates $\sigma(a)$ for single years a are known, then P(a) in Eq. F26 can be replaced by "$\sigma(a)$*P(a)". One could even drop the boundaries B1 and B2 by setting "$\sigma(a)=0$" for birds that have not reached the breeding age.)

3. Types of mortality distributions

So far, it was assumed that the mortality "**M(a)**" is known. In other publications (e.g. [1]), different mortality distributions were considered.

The simplest distribution assumes that all birds reach the same age "**L**", meaning that "**M(L)=1**" and "**M(a)=0**" for "**a≠L**" is a degenerative distribution. Although this distribution simplifies the computations significantly – most of the sums have just one non-zero term – it is quite unrealistic.

A more realistic assumption is that the causes of death for wildlife birds are e.g. predation, shooting, extreme weather, accidents and infections. It is assumed that causes usually strike the birds independent of their age, so that each year a certain proportion of birds dies from all age groups. This leads to a geometric distribution where the mortality has an exponential form "$M(a) \sim x^a$ **(x<1)**". In this case, the yearly mortality is independent of the age.

The parameter "**x**" can either be derived from Eq. F15 or as a function of the proportion of young birds "**g**" and the growth factor "**r**" in Eq. F18.

The problem of this mortality model is that the maximum age is unlimited and permits arbitrary old birds although with a very low probability. Therefore, we will also consider a truncated geometric distribution.

3.1. Degenerative mortality

All birds are assumed to die at age "**a=L**". According to Eqs. F5, F10 and F22, mortality "**M(a)**", survival probability "**S(a)**" and the proportion "**P(a)**" of birds of age "**a**" in the population have the following values.

M(L)=1 and M(a)=0 for a≠L,<br>S(a)=1 for a<L und S(a)=0 for a≥L,<br>$P(a)=g * S(a) * (1+r)^{-a}$ for a<L and P(a)=0 for a≥L.	F27

Based on Eq. F15, the growth rate is then "$r=g*(1+r)-g*(1+r)^{1-L}$"and solving for "**g**" and "**L**", respectively, one obtains

$g(L,r) = \frac{r}{(1+r)} \cdot \frac{(1+r)^L}{(1+r)^L - 1}$ respectively $L(g,r) = 1 - \frac{\ln\left(1 + r - \frac{r}{g}\right)}{\ln(1+r)}$	F28

The equation on the left-hand side computes the proportion of young birds depending on the growth rate "**r**" and the age "**a**" at which all birds die, in this case the maximum age "**L**". The equation on the right-hand side derives the (maximum) age "**L**" from the observed proportion "**g**" of young birds and the (constant) growth rate "**r**".

For a stationary population "**r=0**", these equations lead to a division by zero. But when we take the limit "**r→0**", we obtain the already mentioned relations "**g=1/L**" and "**L=1/g**", respectively.

Formally speaking, "**L**" is assumed to be a positive integer number. Eq. F28 will usually lead to a non-integer number for "**L(g,r)**". This can nevertheless be used in the sense of an approximation or interpolation.

3.2. Geometric distribution (Expo)

When mortality follows a geometric distribution

$M(a)=c*x^a$ for a>0	F29

the parameters “**c**” and “**x<1**” must still be determined. Writing a geometric distribution as in Eq. F29 implies “**c=(1-x)/x**”. Inserting the mortality “**M(a)**” in Eqs. F10 and F12, one obtains the survival probability

$S(a) = M(a+1)+M(a+2)+M(a+3)+ ..... = c*(x^{a+1}+x^{a+2}+x^{a+3}+...) = c * x^a * (x+x^2+x^3+...) = x^a$	F30

and the yearly mortality “**rM(a) = 1-x**”. The average life expectancy is the expected value of the geometric distribution, which is here “**L= 1/(1-x)**”.

Inserting “**S(a)=x^a**” in Eq. F18, yields for the proportion of young birds “**g**”

$$g = \frac{1}{\sum_{a=0}^{\infty} (1+r)^{-a} \cdot x^a} = \frac{1}{\sum_{a=0}^{\infty} \left(\frac{x}{1+r}\right)^a} = \frac{1}{1 + \frac{x}{1+r} + \left(\frac{x}{1+r}\right)^2 + \left(\frac{x}{1+r}\right)^3 + ...} = \frac{1+r-x}{1+r} \quad \text{F31}$$

From the observed proportion of young birds “**g**” and the growth rate “**r**” one can solve this equation for the parameter “**x**” of the geometric distribution, yielding

x = (1-g)*(1+r) and L = 1/(1-x) = 1/(g-r+r*g)	F32

Again, for a stationary population with “**r=0**” we obtain the known relation “**L=1/g**”.

The following box summarizes the formulae for the parameters of interest in this model depending on the observed proportion of young birds “**g**” and the (constant) growth rate “**r**”.

$$L = \frac{1}{g - r + r \cdot g} \qquad M(a) = \frac{(L-1)^{a-1}}{L^a} \qquad S(a) = \left(\frac{L-1}{L}\right)^a \qquad P(a) = g \cdot (1-g)^a$$

$$rM(a) = \frac{1}{L} \qquad A = \frac{1-g}{g} \qquad Le(a) = L \qquad G(B1) = \frac{B1 \cdot g - g + 1}{g} \quad \text{F33}$$

The last breeding age “**B2**” is assumed to be unlimited here.

3.2.1. Application example 1: Average life expectancy “L”

We consider a population with a proportion “**g=0.2=20%**” of young birds. Eq. F33 then yields

r=0 (stationary population):	L=1/g=5 years
r=0.03 (doubling time 23.4 years):	L=5.682 years
r=−0.03 (half-value period 22.8 years):	L=4.464 years

The influence of the growth rate “**r**” is stronger for a geometric mortality distribution in comparison to a degenerated distribution.

3.2.2. Application example 2: Mortality “M(a)”, survival probability “S(a)”, and age distribution “P(a)”

For “**g=0.2**” and “**r=0**” the survival probability for age “**a=25**” years is at the negligible level of 0.4%. The average age “**A**” in the population is 4 years and the generation length “**G**” is 7 years assuming that the first breeding age “**B1**” is at 3 years. The mortality “**M(a)**”, survival probability “**S(a)**”, and age distribution “**P(a)**” are shown in Fig. 1.

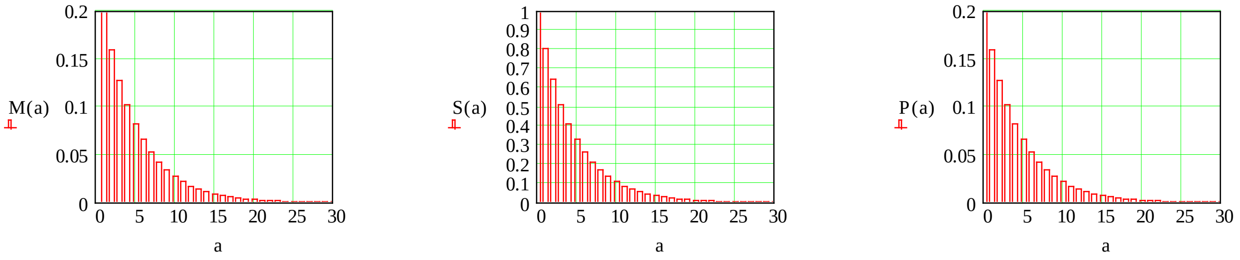

***Fig. 1**: Mortality "**M(a)**", survival probability "**S(a)**", and age distribution "**P(a)**" depending on the age "**a**" for a proportion of young birds "**g=0.2**" corresponding to an average life expectancy of "**L=5**" years (for "**r=0**").*

All age values start at the counting time of the birds not at the hatching time. The average life expectancy "**L**" of hatching birds is smaller because hatchlings and very young birds have a higher mortality compared to older birds. This higher mortality between hatching and counting is not taken into account in all these computations. In the main manuscript we will also consider an example where we take the higher mortality of very young birds into account (Black Redstarts as example).

3.2.3. Application example 3: Varying proportion of young birds

All computations in Eq. F15 assume that both the proportion of young birds "**g**" and the growth rate "**r**" are constant over a longer period of time or can be replaced by their mean values. The following example illustrates what happens when the proportion of young birds "**g**" starts to vary from a certain year on. We assume that "**g**" is constant at the level 0.2 until year 0 and that mortality follows a geometric distribution with an average life expectancy of L=5 years. Starting at year 1, "**g**" starts to vary randomly between 0.1 and 0.3 around the mean value of 0.2. According to Eq. F13, the population change can be computed iteratively after year "**j=0**" where "$\mathbf{M_{i,j} = M(i-j) = M(a)}$" as in Eq. F33. The initial population size in year "**j=0**" is "$\mathbf{N_0=100}$". The population size is computed for the years "**j=0,…,10**". The result depends on the specific random numbers. **Fig. 2** shows an example with quite extreme fluctuations of "**g**".

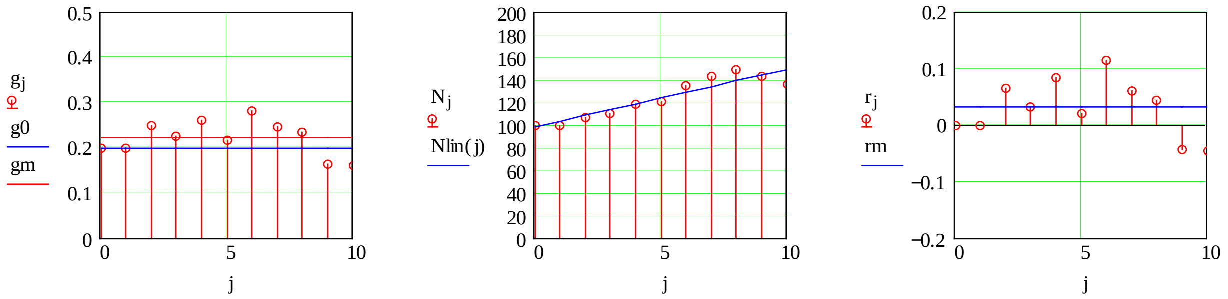

***Fig. 2** Yearly proportion "g_j"of young birds (randomly generated between 0.1 and 0.3 with mean "g_0=0.2"), estimated population sizes "N_j" and growth rates "r_j"changing over the years "j" for a specific configuration of random values "g_j" with "g_m=0.223" as the sample mean of the proportion of young birds, "$N_{lin(j)}$" the linearised population size and "r_m=0.032" the sample mean of "r_j".*

If such fluctuations of "**g**" occur in reality, one should interpret the model with care. Nevertheless, even here the means "$\mathbf{g_m=0.223}$" and "$\mathbf{r_m=0.032}$" are quite close to the "true" value 0.2 and 0, respectively. And the average life expectancy "**L**" is estimated almost correctly with "$\mathbf{L_m=1/(g_m-r_m+g_m*r_m)=5.047}$" with an error of 0.047 years which is negligible in comparison to other uncertainties that will lead to errors in the range of months.

Further investigations in this direction have shown that the model is quite robust against fluctuations of "**g**". Thus, variations of "**g**" can therefore be replaced by the mean value "$\mathbf{g_m}$" without losing much precision. However, systematic changes – e.g. caused by climate change – require further considerations.

3.3. Modified geometric mortality distribution (ModExpo)

The geometric distribution brings two obvious disadvantages.

a) For the first year of life (starting from the counting time) there is no increased mortality considered.
b) The theoretical maximum age is infinite although unrealistic ages will have a very low probability.

Therefore, we modify the geometric distribution and introduce two additional parameters "**M1**" and "$\mathbf{a_{max}}$". The variables that we need to know for our model are now:

- „**g**": proportion of young birds.
- „**r**": yearly population growth.
- „**M1**": Mortality in the first year, i.e. **M1=M(1)**.
- „$\mathbf{a_{max}}$": The maximum possible (or maximum) known age for the birds.

In the range from "**a=2**" to "$\mathbf{a=a_{max}}$" the exponential law "$\mathbf{M(a)=c*x^a}$"is still valid, i.e. that the individual mortality is the same for all age groups except for the first year.

3.3.1. Numerical procedure

This more realistic model does not permit closed form solutions anymore as they were provided for the simpler case in Eq. F33. Therefore, numerical solutions are required. These numerical solutions can for instance be computed using the Excel function "Solver". This module is freely available for Excel, but must be activated first (see the instructions in the Excel file **Supporting Information S2**).

3.3.2. Practical computation with EXCEL

The order of computations is the same as in the examples in Section 3.2.

For the mortality, the constant "**c**" (see Eq. F29) is first calculated from "$\mathbf{M(a)=c*x^a}$" with "**M(0)=0**" and "**M(1)=M1**" and "**M(a)=0**" for "$\mathbf{a>a_{max}}$". Based on the mortality "**M(a)**", the survival probability "**S(a)**" can be computed according to Eq. F10 both depending on the parameter "**x**". This parameter is then estimated based on Eq. F18 given the observed value of "**g**" and "**r**". Then the other relevant variables such as the average live expectancy "**L**", the age distribution "**P(a)**", the yearly mortality "**M(a)**" and others can be calculated.

An example for an Excel sheet is shown in **Fig. 3**.

Input:			Results:						
g=	0.2		g=	0.200	(by Solver)				
r=	0.03		L=	5.622					
M1=	0.2		A=	3.751					
amax=	20		G=	6.458	(from a=3 to a=20))				
x=	0.851								
c=	0.173								
a	x^a	$(1+r)^{-a}$	M(a)	S(a)	rM(a)	$S(a)*(1+r)^{-a}$	P(a)	a*P(a)	Le(a)
0	1.000	1.000	0.000	1.000	0.223	1.000	0.200	0.000	5.622
1	0.851	0.971	0.200	0.800	0.181	0.777	0.155	0.155	5.778
2	0.724	0.943	0.125	0.675	0.182	0.636	0.127	0.254	5.664
3	0.616	0.915	0.106	0.568	0.184	0.520	0.104	0.312	5.538
	…	…	…	…	…	…	…	…	…
	…	…	…	…	…	…	…	…	…
	…	…	…	…	…	…	…	…	…
17	0.064	0.605	0.011	0.024	0.406	0.015	0.003	0.050	1.893
18	0.055	0.587	0.009	0.015	0.554	0.009	0.002	0.031	1.460
19	0.046	0.570	0.008	0.007	1.000	0.004	0.001	0.015	1.000
20	0.040	0.554	0.007	0.000	#BEZUG!	0.000	0.000	0.000	0.000
Sum:	4.627		1.000	5.622		5.000	1.000	3.751	
				= L		= 1/g?		= A	

Fig. 3: *Excel sheet "modExpo-M1-amax.xlsx" with input values "**g**", "**r**", "**M1**" and "$\mathbf{a_{max}}$"in blue bold-faced font (upper left) and the most important results in red bold-faced font on the right-hand side. The age groups from "**a=4**" to "**a=16**" are omitted due to limited space (for details see text).*

The Excel sheet is based on the following virtual population. The input values are:

- Maximum age of 20 years "$\mathbf{a_{max}=20}$", leading to the entries 0-20 in the column "**a**".
- Assumed (observed) proportion of young birds "**g=0.2**".
- Yearly growth rate "**r=0.03**".
- Mortality in the first year: 20% with "**M1=0.2**".

The constant “**c**” is the normalisation factor guaranteeing that the sum of mortalities is 1. The value for “**x**” must be defined by the user so that Eq. F18 is satisfied. Otherwise there will be a mismatch between the computed “**g-value (red)**” and the given “**g-value (blue)**”. The value for “**x**” can always be found by a simple trial and error procedure or – a better option – by using the Excel "Solver".

The results for the simulation are:

- Survival probability “**S(a)**”.
- Normalised age distribution “**P(a)**“ where “**P(0)=g**”
- Average residual life expectancy “**Le(a)**” for birds of age “**a**”.
- Yearly (relative) mortality “**rM(a)**” as defined in Eq. F12.

(The Excel program with short instructions is available as **Supporting Information S2**)

The most important results (in red bold-faced font) are the average life expectancy “**L**” (excluding the time after hatching and before counting), the average age “**A**” in the population and the mean generation length “**G**”. The simple model based on the unmodified geometric distribution based on “**g=0.2**” and “**r=0.03**” would yield “**L=5.682**” (instead of 5.622), “**M1=0.176**” (instead of 0.2), “**A=4**” (instead of 3.751) und “**G=7**” (instead of 6.458) according to Eq. F33. The differences are mainly caused by the increased mortality “**M=0.2**” in the modified geometric distribution (instead of “**M=0.176**”) and partly by the truncation at the maximum age “$\mathbf{a_{max}=20}$”. Taking the general uncertainties caused by data collection into account, these differences are more or less negligible.

Larger differences between the geometric distribution and its truncated version in Eq. F33 can only be expected when the survival probability after the assumed maximum age “$\mathbf{a_{max}}$” is still significantly larger than zero in the unmodified geometric distribution. In this case, the unmodified geometric distribution could lead to birds of an unrealistic old age with a non-negligible probability. In the above example, we would have a small probability of “**S(20)=0.0208**” for the unmodified geometric distribution, which has a negligible influence on the population parameters computed in Eq. 33. In such cases, the additional effort to utilize the modified geometric mortality distribution might be not worthwhile. For birds with a longer life expectancy (e.g. Common Cranes), larger differences are possible.

3.4. Bootstrapping and calculations based on R

3.4.1. Background information bootstrapping

Bootstrapping is performed to test how reliable the mean value of the observed proportion of young birds is over a period of time. First, the mean value of the observed proportion of young birds “**g**” is calculated based on the input file with the counted birds per year (found as an example for the Black-headed gull in **Supporting Information S4**). The program ModExpo needs in addition to the value of “**g**”, the values for the growth rate per year “**r**”, the mortality in the first year “**M1**”, the maximum (known) possible age depending on the species “$\mathbf{a_{max}}$”, the first (mean) breeding age “**B1**” which are specified by the user. Then, the bootstrapping length “**n**” – the number of bootstrap samples – is defined with n=10,000 and the confidence level “**ci**” with a value of ci=95 %. But the number of bootstraps and the confidence level of the confidence interval can be adjusted if necessary. For the probability within the bootstrapping, the number of young birds and the corresponding number of total (young and adult) birds in each year is determined. The bootstrapping leads to a simulation of the parameters for the average life expectancy “**L**”, the mean age in a population “**A**”, and the generation length “**G**”. For each of the parameters the specific confidence interval is determined as the corresponding quantiles of the parameters computed in the bootstrap samples.

A single bootstrap sample is generated in the following way. For each year the specified number of juvenile and adult birds is taken as set from which a sample of the same size is drawn with replacement. This leads to a bootstrap sample similar to the original data table with random deviations. For each of the bootstrap samples the parameters for the average life expectancy “**L**”, the mean age in a population “**A**”, and the generation length “**G**” are calculated.

3.4.2. Instructions for using the “ModExpo” method in R

This method is referred to Chapter 3.3 "Modified geometric mortality distribution (ModExpo)". It uses the formulas described in Chapter 2 "Background for the computations". The method is implemented in R and the input variables are as follows:

- Observed proportion of young birds “**g**“,
- Yearly population growth rate “**r**“,
- Mortality in the first year, i.e. “**M1=M(1)**”; “M1=0 means “auto”
- The maximum possible (or maximum) known age for the bird species “$\mathbf{a_{max}}$”,
- First (mean) breeding age, “**B1**”

The open source statistics software R (see https://www.r-project.org/) must be installed on the computer. The R file provided (**Supporting Information S3**) can be opened with any text editor. At the top of the file, values for the above parameters are given (as example for the Black-headed Gull with “**r=0**”, “**M1=0**”, “$\mathbf{a_{max}=30}$”, and “**B1=3**”). These parameters should be changed for other bird species!

Once these parameters are adjusted, one can simply copy and paste the full content of the text file to the R console. It should be noted that the R-package "readxl" will be installed requiring internet connection. This package needs only to be installed the very first time the program is running.

R will then open a file reader window in which the desired Excel file with the bird counts can be selected. The file must be in the same format as the example file (**Supporting Information S4**, including the counts of young and adult birds with the example of the Black-headed Gull).

After the calculation, all results are displayed on the console in R. First, the above mentioned determined values of the input file are shown and then the input values specified by the user. The input values “**x**” and “**c**” are calculated and needed to have very close values of the entered or file-specific value “**g**” and the calculated value “**g**” by “ModExpo”. The results of “ModExpo” are observed proportion of young birds “**g**” (should be very close the input value “**g**”), the average life expectancy “**L**”, the mean age in a population “**A**” and the generation length “**G**”. If applying bootstrapping, the 95%-confidence intervals are shown for the values “**L**”, “**A**”, and “**G**” based on the defined bootstrapping length. Additionally, some control values are shown where the values of “Sum of M(a)” and “Sum of P(a)” have to be very close to 1.

3.5. Other approaches and further perspectives

In earlier publications ([1-3]) other models for population dynamics were investigated based on ideas from mortality tables where mixtures of exponential and normal distributions are considered. The general procedure was always the same. Once the mortalities M(a) with at least one free parameter were specified, the free parameter or parameters was or were determined based on Eqs. F15 or F18, given the proportion “**g**” of young birds and the growth rate “**r**”. All other parameters of interest can then be calculated using Eqs. F15 - F26. The average life expectancies “**L**” were always between the extreme of the degenerated distribution in Section 3.1 and the (unmodified) geometric distribution in Section 3.2.

The drawback of these earlier approaches was mainly the higher computational effort.

Another possible approach could model mortality “**M(a)**” by a function that leads to a linearly decreasing survival probability “**S(a)**” instead of an exponential one. **Fig. 4** shows an example with a proportion “**g=0.2**” of young birds, a stationary population “**r=0**” and a maximum age of “$\mathbf{a_{max}=9}$” years. The maximum age “$\mathbf{a_{max}}$” was determined in such a way that Eq. F18 yields the given value of “**g**”.

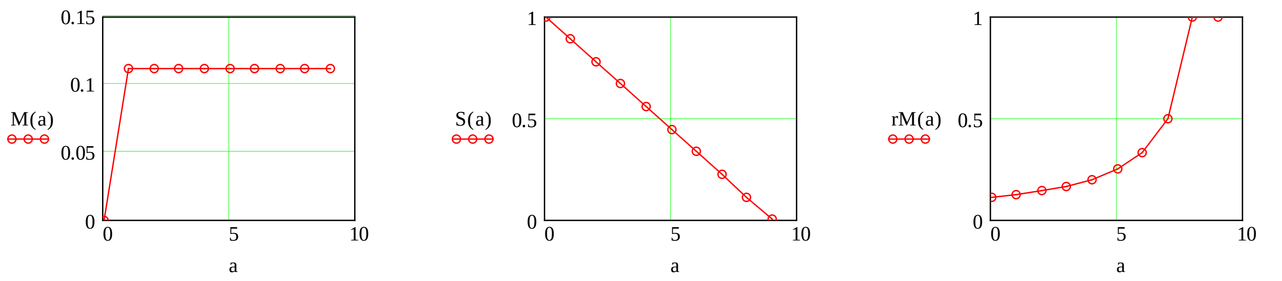

Fig. 4: *Mortality “**M(a)**”, survival probability “**S(a)**” and yearly mortality “**rM(a)**” depending on the age “**a**” (here from 0 to 10) for a proportion of young birds “**g=0.2**”, a growth rate of “**r=0**”, and a maximum age of 9 years “$\mathbf{a_{max}=9}$”.*

For this linear survival probability “**S(a)**”, the yearly (relative) mortality “**rM(a)**” increases with age. This would be plausible when all or the majority of birds would reach the biological maximum age “$\mathbf{a_{max}}$”. However, wildlife birds seldom reach this maximum age “a_{max}” because they usually die before (e.g. predation, accidents, diseases). Therefore, the exponential survival probability might be a more realistic model consistant with a constant mortality rate independent of the age (except for the higher first year mortality).

More complex mortality models for the limited setting based on bird counts might be a sledgehammer to crack a nut. At least for birds with a limited life span, the simple unmodified geometric mortality distribution with the simple formulae given in Eq. F33 should suffice. In any stationary population **"L=1/g"** always holds.

4. References

[1] Oldekop, W. (2011): Wie lange leben unsere Kraniche? - eine populationsdynamische Parameterstudie zur Lebenserwartung von Vögeln. AVES Braunschweig 2: 45-56

[2] Oldekop, W. (2014): Zur Lebenserwartung freilebender Vögel. AVES Braunschweig 5: 39-53

[3] Rinas, U. & W. Oldekop (2017): Altersdifferenzierte Zufallsbeobachtungen der Lachmöwe (*Larus ridibundus*) - Erkenntnisse zu Lebenserwartung, Bruterfolg und Alterssegregation. AVES Braunschweig 8: 38-52
